# The effect of comorbidities on glycemic control among Colombian adults with diabetes mellitus: a longitudinal approach with real-world data

**DOI:** 10.1186/s12902-021-00791-w

**Published:** 2021-06-26

**Authors:** Manuel Urina-Jassir, Lina Johana Herrera-Parra, Juliana Alexandra Hernández Vargas, Ana María Valbuena-García, Lizbeth Acuña-Merchán, Miguel Urina-Triana

**Affiliations:** 1 Fundación del Caribe para la Investigación Biomédica, Carrera 50 # 80 – 216 Office 201, Barranquilla, Atlántico Colombia; 2 Cuenta de Alto Costo, Fondo Colombiano de Enfermedades de Alto Costo, Carrera 45 # 103 - 34, Bogotá, D.C Colombia; 3 Facultad de Ciencias de la Salud, Universidad Simón Bolívar, Carrera 59 # 59 – 65, Barranquilla, 080002 Colombia

**Keywords:** Glycated hemoglobin A1c, Diabetes mellitus, Comorbidity, Hypertension, Chronic kidney Disease, Obesity

## Abstract

**Background:**

Achieving an optimal glycemic control has been described to reduce the incidence of diabetes mellitus (DM) related complications. The association between comorbidities and glycemic control remains unclear. Our aim is to evaluate the effect of comorbidities on glycemic control in people living with DM.

**Methods:**

A retrospective longitudinal study on data from the National Registry of Chronic Kidney Disease from 2014 to 2019 in Colombia. The outcome was poor glycemic control (PGC = HbA1c ≥7.0%). The association between each comorbidity (hypertension (HTN), chronic kidney disease (CKD) or obesity) and PGC was evaluated through multivariate mixed effects logistic regression models. The measures of effect were odds ratios (OR) and their 95% confidence intervals (CI). We also evaluated the main associations stratified by gender, insurance, and early onset diabetes as well as statistical interaction between each comorbidity and ethnicity.

**Results:**

From 969,531 people at baseline, 85% had at least one comorbidity; they were older and mostly female. In people living with DM and CKD, the odds of having a PGC were 78% (OR: 1.78, CI 95%: 1.55-2.05) higher than those without CKD. Same pattern was observed in obese for whom the odds were 52% (OR: 1.52, CI 95%: 1.31-1.75) higher than in non-obese. Non-significant association was found between HTN and PGC. We found statistical interaction between comorbidities and ethnicity (afro descendant) as well as effect modification by health insurance and early onset DM.

**Conclusions:**

Prevalence of comorbidities was high in adults living with DM. Patients with concomitant CKD or obesity had significantly higher odds of having a PGC.

**Supplementary Information:**

The online version contains supplementary material available at 10.1186/s12902-021-00791-w.

## Background

Diabetes Mellitus (DM) prevalence is increasing worldwide [1]. The International Diabetes Federation estimated that 9.3% (463 million people) of the global adult population had DM in 2019; with an unsettling projection of 10.2% (578 million people) and 10.9% (700 million people) for 2030 and 2045, respectively [1]. The same phenomena was projected for the South and Central American region; with a prevalence up to 8.5% (31.6 million people) and a prediction up to 9.9% (49.1 million people) in 2045 [1]. The aforementioned region includes Colombia, a country where DM prevalence ranges from 1.8-11.2% depending on the report consulted [2]. This is alarming given the effects DM has on morbidity and the sustainability of health systems [3].

Achieving an optimal glycemic control has been described to decrease the incidence of DM related complications [4–7]. As for microvascular complications, in a recent meta-analysis a more rigorous control was found to reduce the relative risk by 20 and 13% for kidney and eye outcomes, respectively [5]. On the other hand, an effect on macrovascular complications was also described with a reduction in cardiovascular events when intensive glycemic control is employed [6, 7]. In spite of this, only few patients reach the established target; a meta-analysis described that approximately 36% of patients in randomized control trials and 34% in cross-sectional studies achieved the hemoglobin A1c (HbA1c) target (≤7.0% or < 7.0%, respectively) [8].

Different sociodemographic and clinical factors appear to be associated with glycemic control. Regarding age and gender, female and younger patients were more commonly associated with poor control [9]. Taking into account race and ethnicity, studies have demonstrated significant differences between ethnic groups in achieving glycemic target [10, 11]. An additional determinant of inadequate glycemic control is a longer time since the diagnosis of DM [9, 12].

Comorbid conditions, mainly hypertension (HTN), obesity, hyperlipidemia, chronic kidney disease (CKD) and cardiovascular disease, are common in patients living with DM [13]. Studies evaluating the relationship between the presence of comorbidities and glycemic control have showed contradictory findings depending on the analysis. When the total number of comorbidities was used, studies have found no relationship [14] or an inverse relationship [15] between comorbidities and HbA1c. Research using comorbidity scores also established no relationship with HbA1c levels [16]. Whereas in a study evaluating specific comorbidities, subjects with HTN and dyslipidemia were found to have lower odds of achieving glycemic target while no significant association was observed with CKD [17]. Lastly, patients living with DM and overweight or obesity were more commonly associated with poor glycemic levels [18].

Considering the detrimental effects of suboptimal glycemic control, the identification of patients at an increased risk of PGC is crucial for health systems and policy makers as an aid to develop strategies to capture at-risk individuals. Therefore, we aimed to assess the longitudinal effect that common DM-related comorbidities (HTN, CKD, and obesity) have on glycemic control among Colombian adults diagnosed with DM from 2014 to 2019.

## Methods

### Data sources

We conducted a retrospective longitudinal analysis on data of people diagnosed with DM who were reported by health insurers and providers to the National Registry of Chronic Kidney Disease (NRCKD) from July 1st, 2013 to June 30th, 2019 in Colombia. The NRCKD is managed by the High-Cost Diseases Fund (“Cuenta de Alto Costo” in Spanish). It has been operating since 2008 by a resolution from the Ministry of Health of Colombia [19]. The NRCKD aims to evaluate the burden of CKD and its most common precursors (HTN and DM) as well as the effective access to health services related to prevention, diagnosis and control across the country.

The NRCKD is an administrative and passive registry with a national scope because approximately 98% of the population is affiliated to the national healthcare system [20], and their insurers are required to report patients living with HTN, DM or CKD to the registry [19]. A unique identification number is assigned to each individual protecting their personal information and allowing future follow up. When a new case enters the NRCKD, a complete registration is done; for old cases, data are updated every year.

The NRCKD undergoes a data auditing process to ensure the veracity of the information. The first step involves the use of an algorithm to identify mistakes in the reporting process. After this, an experienced team compares the reported information with health clinical records by a well-stablished data monitoring process in a representative stratified sample of cases with HTN and/or DM, with or without CKD. In case of any inconsistency is identified, the real data on clinical records is captured.

### Eligibility of participants

**Fig. 1:**
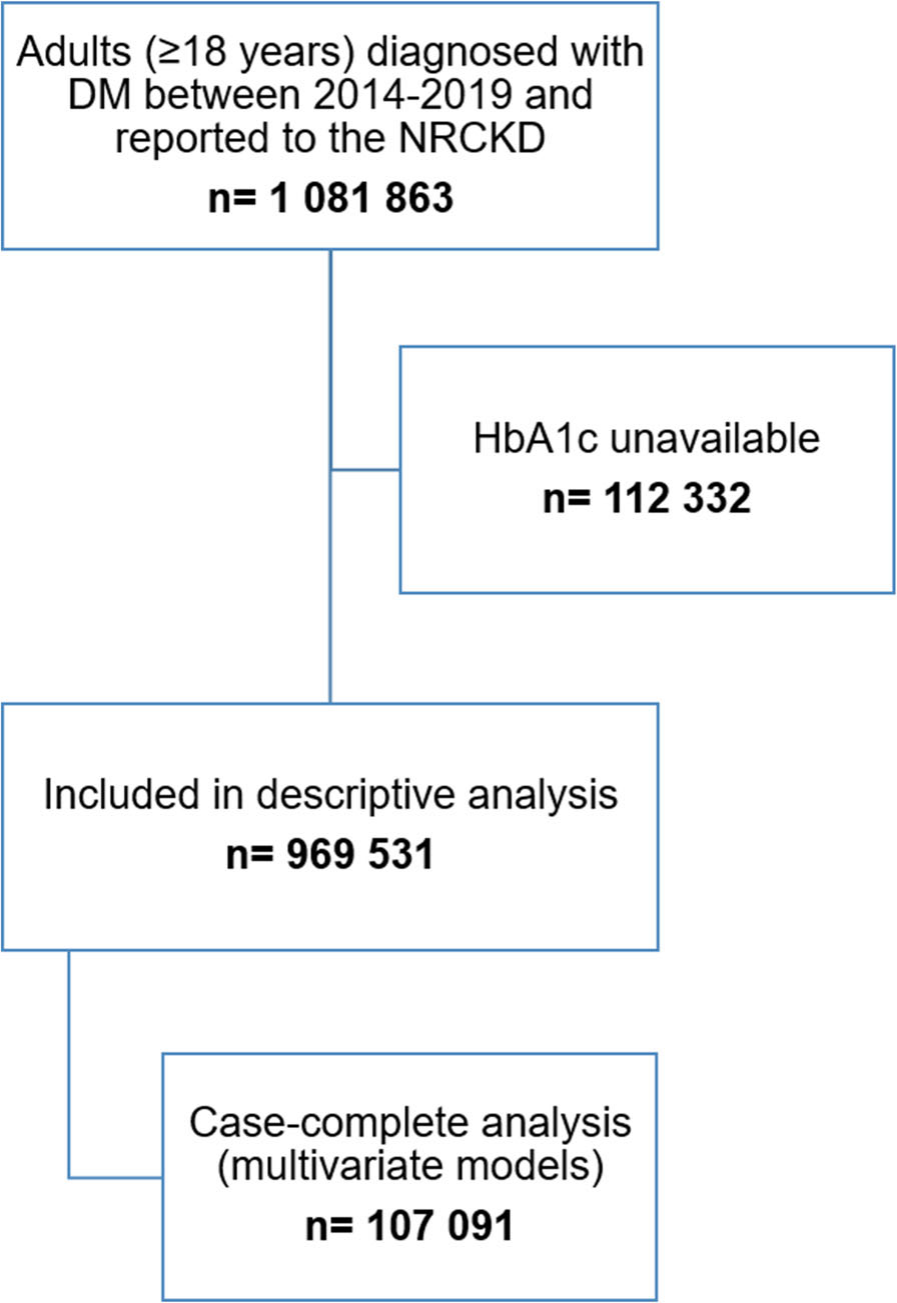
Eligibility flow of studied population from the National Registry of Chronic Kidney Disease, Colombia 2014-2019. Abbreviations: DM: diabetes mellitus, NRCKD: National Registry of Chronic Kidney Disease, HbA1c: glycated hemoglobin

A total of 1,081,863 people aged 18 years or more, diagnosed with DM were reported to the NRCKD from 2014 to 2019. 112,332 were excluded because they had no information available on the measurement of HbA1c. Indeed, 969,531 people met the inclusion criteria and were analyzed. Figure 1 shows the eligibility flow of the population studied.

### Exposure and outcome variables

The presence of comorbidities (HTN, CKD, obesity), reported by health insurers to the NRCKD was considered as the exposure of interest. HTN diagnosis was reported to the NRCKD as its presence or absence. This diagnosis is defined by the treating physician based on his/her clinical judgement [21, 22]. CKD diagnosis was defined by the patients’ treating physician and reported to the NRCKD by their insurers as follows: yes/no/undetermined/not studied for CKD during the reporting period. CKD diagnosis was verified by a data monitoring process following an algorithm based on the Colombian CKD guideline that defines the presence of CKD as abnormalities in the structure or function (GFR < 60 ml/min/1.73 m^2^) for more than 3 months [23]*.* People classified as undetermined were those with early kidney impairment that require evidence of functional or structural injury for more than 3 months. Lastly, obesity was determined using the World Health Organization definition (body mass index [BMI] ≥30 kg/m2) [24]. Exhaustiveness and accuracy of exposures were verified in clinical records by a well-established data monitoring process.

A poor glycemic control (PGC) was the dependent variable. It was defined by the measurement of HbA1c, reported during the last 6 months of each follow up period. PGC was treated as a dichotomous variable for each point in time using the cutoff of HbA1c ≥ 7.0%, according to the standard defined by the American Diabetes Association [25].

### Covariates

Demographic information included age, gender, health insurance (third payer, state insurance, special insurance, exception insurance or uninsured), and region of residence following the Administrative Department for National Statistics (“Departamento Administrativo Nacional de Estadística” in Spanish) classification (Bogotá, D.C., Central, Eastern, Pacific, Caribbean and Amazonian) [26] (Additional File 1: Table S1). Ethnicity was self-reported and classified in indigenous, afro descendant and other (white, mestizo, gypsy or not classified). In respect of clinical data, age at diagnosis of DM and comorbidities were included. Reported information on weight (kg) and height (meters) was used to calculate BMI (kg/m^2^). Otherwise, glomerular filtration rate (GFR) was estimated using the CKD-EPI method and expressed as mL/min/1.73m^2^ [27]. On the other hand, CKD stages were defined based on the GFR as follows: 1: GFR > 90 with clinical evidence of kidney damage; 2: GFR: 60-90 with clinical evidence of kidney damage; 3: GFR: 30-60, 4: GFR 15-30, 5: GFR: < 15 [28]. Lastly, DM duration was calculated using the date of last contact (last HbA1C test) and the date of diagnosis and informed in years.

### Statistical analysis

#### Descriptive analysis

Baseline demographic and clinical characteristics of the cohort are presented as means or medians and their standard deviation (SD) or interquartile range (IQR), according to their distribution, evaluated by statistical and graphical methods. Otherwise, qualitative data is informed as absolute values (proportions). Baseline characteristics were compared across comorbidities (HTN, CKD and obesity) by using a X^2^ test for categorical data and Wilcoxon test was used for numeric variables. We also calculated absolute standardized differences (ASD) between groups of comorbidities to avoid limitations of hypothesis tests taking into account the effect of the sample size. In the case of continuous variables with skewed distribution, rank statistics were used instead the mean [29]. ASD > 10% were considered significant.

#### Assessment of HbA1c trend

Furthermore, the trend of variation on HbA1c levels during the follow-up for patients with different baseline comorbidity profiles was evaluated by estimating generalized linear mixed models assuming random intercepts for each participant. An interaction term between HbA1c levels and time was included to take into account variations over time, adjusting by each comorbidity. From each model, the predicted Hb1Ac values were plotted over time.

#### Evaluation of the association between comorbidities and poor glycemic control

The association of interest was estimated using a mixed effects logistic regression model with aleatory intercept, which modelates the odds of PGC for each point in time, taking into account the effect of repeated measures and considering the parameters related to the intercept differentially between each participant, but with a constant slope. The final model for each comorbidity was adjusted by age, gender, ethnicity, health insurance, duration of diabetes. In case of HTN and CKD we also included the BMI (time variant) as a confounder in the final models. Effect measures were the odds ratios and their 95% confidence intervals. We also repeated the final models stratifying by gender, health insurance, and early onset DM (diagnosis < 45 years old [30]) to evaluate a potential effect modification.

#### Estimation of interaction models

Furthermore, the interaction between each comorbidity and intermediate variables such us ethnicity was explored by the inclusion of a multiplicative term in the final models. *p* values < 0.05 (two-tailed) were considered statistically significant, except for the effect modification model (*p*-value < 0.20).

#### General considerations

Trend and association models were estimated using a case complete approach including people with 6-year HbA1c measurement (*n* = 107,091). All statistical analyses were performed in Stata version 13 (StataCorp LP, College Station, Texas, USA) and graphs were created in R version 4.0.2.

### Sensitivity analysis

We performed sensitivity analysis including a BMI ≥25 kg/m^2^ instead of ≥30 kg/m^2^ as the exposition to verify the consistency of the association obtained in the main model. Furthermore, we evaluated the association in people with CKD by using a different cut off to define the outcome (HbA1c ≥ 8.0%). Taking into account the potential effect of early onset on PGC, it was tested as a covariate in the final models.

## Results

### Demographic and clinical characteristics at baseline

969,531 people were analyzed. Mean age at baseline was 61.4 years (SD 13.2), 58.1% were female and 75.7% had third payer insurance. Most people (29.5%) lived in the Central region, followed by Bogotá, D.C. (20.3%). Around 96.0% were self-recognized as “other” ethnicity and 3.5% were afro descendant. Mean age at DM diagnosis was 59.2 years (SD 13.0), median of DM duration was 2.76 years (IQR 1.0-6.0), 56.8% had DM evolution < 5 years and 9.9% had early onset DM. Prevalence of HTN, CKD and obesity were 71.5, 30.6 and 35.2%, respectively. Majority of the people with CKD were classified in stages 2 (43.9%) and 3 (28.9%).

Table 1 shows a characterization by comorbidities. Only 14.9% did not have an associated comorbidity at baseline. Prevalence of comorbidities was higher in women. People with comorbidities were older, except for those with obesity. Age at DM diagnosis was also higher in people with comorbidities. ASD were significant for DM duration in all groups, except for the obese. When comparing the prevalence of obesity by comorbidities, it was also higher in people with HTN and CKD.

**Table 1:** Baseline characteristics of the population studied according to comorbidities in Colombia 2014-2019

Variables^a^		Comorbidities^d^											
	Total (*n* = 969,531)	DM only			HTN			CKD			Obesity		
		Yes (*n* = 144,910)	No (*n* = 824,621)	ASD^e^ (%)	Yes (*n* = 693,015)	No (*n* = 276,516)	ASD^e^ (%)	Yes (*n* = 297,198)	No (*n* = 672,333)	ASD^e^ (%)	Yes (*n* = 341,592)	No (*n* = 627,435)	ASD^e^ (%)
*Age (y)*	61.4 (13.2)	55.0 (13.0)	62.5 (13.0)	58.5	64.0 (12.4)	55.0 (13.0)	71.5	65.1 (14.0)	60.0 (12.6)	40.1	59.4 (12.7)	62.4 (13.4)	23.0
*Female*	563,687 (58.1)	70,424 (48.6)	493,263 (60.0)	22.6	420,943 (60.7)	142,744 (51.6)	18.4	420,943 (57.6)	142,744 (59.2)	3.1	346,158 (55.7)	217,094 (63.5)	17.1
*Health insurance*													
Third payer	734,398 (75.7)	108,856 (75.1)	625,542 (76.0)	3.1	528,788 (76.3)	20,561 (74.3)	5.4	217,168 (73.0)	517,230 (77.0)	9.2	271,457 (79.4)	462,723 (73.7)	13.6
State	230,448 (23.8)	35,529 (24.5)	194,919 (23.6)		160,578 (23.1)	69,870 (25.2)		78,500 (26.4)	151,948 (22.6)		68,564 (20.0)	161,600 (25.7)	
Special	1946 (0.2)	319 (0.2)	2312 (0.3)		1576 (0.2)	370 (0.1)		486 (0.1)	1460 (0.2)		687 (0.2)	1258 (0.2)	
Exception	2631 (0.2)	202 (0.1)	1744 (0.2)		1988 (0.2)	643 (0.2)		982 (0.3)	1649 (0.2)		853 (0.2)	1777 (0.3)	
Uninsured	108 (0.1)	4 (0.0)	104 (0.01)		85 (0.1)	23 (0.1)		62 (0.2)	46 (0.1)		31 (0.1)	77 (0.1)	
Region of residence													
Bogotá, D.C.	196,815 (20.3)	32,666 (22.5)	164,149 (20.0)	17.1	134,398 (19.4)	62,417 (22.5)	16.5	55,182 (18.5)	141,633 (21.0)	20.2	72,354 (21.1)	124,389 (20.0)	8.2
Caribbean	168,465 (17.4)	29,317 (20.2)	139,148 (17.0)		117,582 (17.0)	50,883 (18.4)		44,985 (15.1)	123,480 (18.3)		53,045 (15.5)	115,317 (18.3)	
Eastern	131,918 (13.6)	21,638 (15.0)	110,280 (13.3)		89,233 (13.0)	42,685 (15.4)		40,145 (13.5)	91,773 (13.6)		48,960 (14.3)	82,882 (13.2)	
Central	286,454 (29.5)	38,127 (26.3)	248,327 (30.1)		215,779 (31.1)	70,675 (25.5)		84,402 (28.4)	202,052 (30.0)		102,160 (30.0)	184,124 (29.3)	
Pacific	178,174 (18.4)	21,415 (14.8)	156,759 (19.0)		131,532 (19.0)	46,642 (17.0)		70,492 (23.7)	107,682 (16.0)		62,450 (18.2)	115,652 (18.4)	
Amazonian	7705 (0.8)	1747 (1.21)	5958 (0.7)		4491 (0.6)	3214 (1.1)		1992 (0.6)	5713 (0.8)		2623 (0.7)	5071 (0.8)	
Ethnicity^b^													
Indigenous	5078 (0.5)	1275 (1.0)	3803 (0.4)	5.6	2883 (0.4)	2195 (0.8)	6.9	1427 (0.5)	3651 (0.5)	16.1	1174 (0.3)	3892 (0.6)	5.3
Afro descendant	34,514 (3.5)	4608 (3.2)	29,906 (3.6)		22,852 (3.3)	11,662 (4.2)		17,220 (5.8)	17,294 (2.5)		10,765 (3.2)	23,730 (3.8)	
Other	929,939 (96.0)	139,027 (96.0)	790,912 (96.0)		667,280 (96.2)	262,659 (95.0)		278,551 (93.7)	651,388 (97.0)		329,653 (96.5)	599,813 (95.6)	
*Age at DM diagnosis (y)*	59.2 (13.0)	53.2 (13.0)	60.3 (12.7)	55.3	61.7 (12.1)	53.1 (13.0)	68.3	62.2 (13.5)	58.0 (12.4)	33.1	63.5 (17.1)	57.4 (12.3)	22.0
*DM duration (y)*^c^	2.76 (1.0-6.0)	1.8 (0.5-4.3)	3.0 (0.9-6.0)	25.5	3.1 (1.0-6.3)	2.0 (0.5-4.3)	37.7	3.2 (1.1-6.6)	2.5 (0.7-5.4)	21.3	2.7 (1.0-5.6)	3.0 (1.0-6.1)	4.3
*BMI (kg/m*^*2*^*)*^c^	28.1 (25.3-31.5)	26.0 (24.0-28.0)	28.7 (25.7-32.2)	83.3	28.4 (25.5-32.6)	27.4 (25.1-30.7)	19.6	27.3 (24.5-30.6)	28.5 (25.7-32.3)	25.2	33.1 (31.2-35.7)	26.2 (24.1-28.1)	32.2
*HTN diagnosis*	693,015 (71.5)	NA	693,015 (84.0)	NA	NA	NA	NA	229,972 (33.1)	67,226 (24.3)	19.2	259,533 (76.0)	433,196 (69.0)	15.5
*Glomerular filtration rate (mL/min/1.73 m)* ^*c,f*^	79.1 (21.5)	77.1 (21.2)	NA	NA	75.4 (20.8)	87.3 (20.0)	57.3	70.3 (23.8)	NA	NA	77.3 (24.8)	77.4 (21.5)	12.4
*CKD diagnosis*	297,198 (30.6)	NA	297,198 (36.0)	NA	229,972 (33.1)	67,226 (24.3)	19.6	NA	NA	NA	86,599 (25.3)	210,361 (33.5)	18.0
*CKD stages* ^c^													
1	65,644 (22.4)	NA	65,644 (22.3)	NA	38,594 (17.0)	27,050 (41.2)	67.0	65,644 (22.4)	NA	NA	24,404 (28.5)	41,181 (20.0)	21.3
2	128,930 (43.9)		128,930 (44.0)		100,570 (44.1)	28,360 (43.1)		128,930 (44.0)			35,281 (41.2)	93,544 (45.0)	
3	84,639 (28.9)		84,639 (29.0)		75,879 (33.3)	8760 (13.4)		84,639 (28.8)			22,326 (26.1)	62,263 (30.0)	
4	7522 (2.6)		7522 (2.5)		6907 (3.1)	615 (1.0)		7522 (2.6)			2212 (2.5)	5307 (2.5)	
5	6571 (2.2)		6571 (2.2)		5683 (2.5)	888 (1.3)		6571 (2.2)			1304 (1.5)	5258 (2.5)	
*Obesity (BMI > 30 kg/m*^*2*^*)* ^*c*^	341,592 (35.2)	NA	341,592 (41.4)	259,533 (37.4)		82,059 (29.7)	16.4	254,993 (38.4)	86,599 (29.1)	18.6	NA	NA	NA

*Abbreviations*: *DM* diabetes mellitus, *HTN* hypertension, *CKD* chronic kidney disease, *BMI* body mass index

^a^ Values are absolute values (percentages) for categorical variables. In case of numeric variables, they correspond to mean (SDs) or median (IQRs)

^b^ Other category includes people self-recognized as white, mestizo, gypsy or not classified

^c^ Less than 5% missing values, except for DM duration in which missing data ranged from 14 to 25%

^d^ Differences between groups were evaluated by using X^2^ and Wilcoxon tests for categorical and numeric data, respectively. All *p*-values were < 0.001

^e^ Absolute standardized difference (ASD), values > 10% were considered significant

^f^ Glomerular filtration rates reported are from patients with CKD diagnosis

### Trends of longitudinal variation on HbA1c by comorbidities

**Fig. 2:**
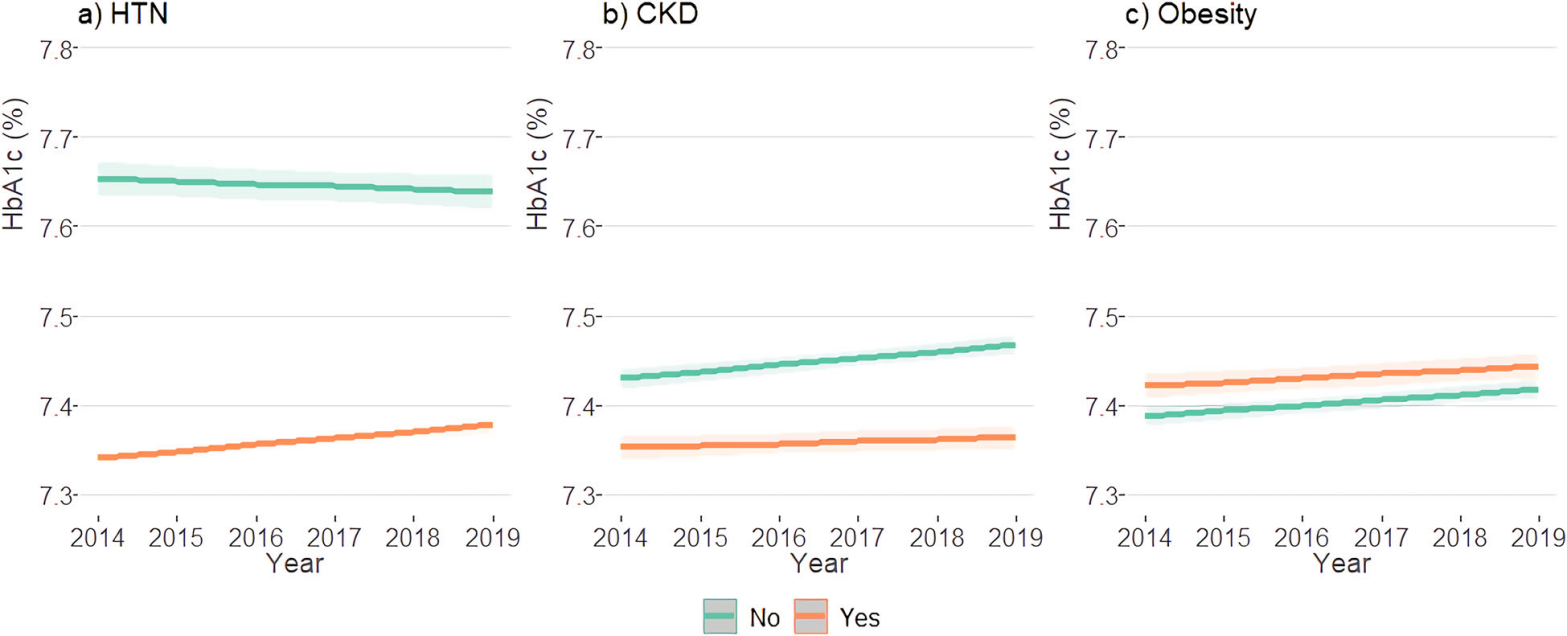
Longitudinal trend of glycated hemoglobin by comorbidities. Colombia 2014-2019. Figure 2 shows the variation on HbA1c over time by comorbidities. In general, there was a pattern on HbA1c levels in people with diabetes and HTN (**a**) or CKD (**b**), characterized by higher HbA1c in those without the comorbidities. However, an inverse trend was observed in obese (**c**). Abbreviations: HTN: hypertension, CKD: chronic kidney disease, HbA1c: glycated hemoglobin

After a 6-year follow up, people living with DM and HTN or CKD had a better glycemic control (lower HbA1c levels) than those with obesity. In both DM subjects with HTN or CKD, HbA1c levels were lower in those who had the condition. These findings were contrary to the obese/not obese subjects, where we found higher HbA1c levels when obesity was present. A higher gap in the HbA1c levels between the presence or not of comorbidities was observed in people living with DM and HTN/no HTN than in the other groups. When estimating the longitudinal variation on HbA1c when changing the cut-off from BMI ≥ 30 kg/m^2^ to 25 kg/m^2^, the trend was opposite to the observed in obese, with a better glycemic control in those with a BMI ≥ 25 kg/m^2^ (Fig. 2).

### Longitudinal association between comorbidities and glycemic control

The mean number of HbA1c measurements was 3.18 (SD 1.7). Only 11.0% of the patients had 6-year HbA1c measurements and were included in the final models. Global prevalence of PGC was 52.7% (95% CI: 52.6 a 52.8). When analyzing the effect of comorbidities on PGC (HbA1c ≥7.0), we observed no significant association between HTN and PGC. Nevertheless, CKD and obesity were significant and directly associated with PGC in all models. In subjects living with DM and CKD, the odds of having a PGC were 78.0% (OR: 1.78, CI 95%: 1.55-2.05) higher than those without CKD. We also observed a strong direct association among people living with DM and obesity, in whom the odds were 52.0% (OR: 1.52, CI 95%: 1.31-1.75) higher than in non-obese (Table 2).

**Table 2:** Multivariate-adjusted odds of poor glycemic control by comorbidities in Colombian adults with diabetes mellitus, 2014-2019

Model	Hypertension			Chronic kidney disease			Obesity		
	OR	95% CI	***p***-value	OR	95% CI	***p***-value	OR	95% CI	***p***-value
Age adjusted	1.08	0.91 - 1.28	0.36	1.76	1.54 - 2.02	< 0.01	1.49	1.30 - 1.71	< 0.01
Multivariable 1 ^a^ (Fixed BMI)	1.08	0.91 - 1.28	0.36	1.75	1.53 - 2.00	< 0.01	1.46	1.27 - 1.68	< 0.01
Multivariable 1 ^b^ (Time variant BMI)	0.98	0.83 - 1.16	0.84	1.87	1.64 - 2.14	< 0.01	NA	NA	NA
Multivariable 2 ^c^ (Fixed BMI)	1.04	0.87 - 1.24	0.66	1.67	1.45 - 1.93	< 0.01	1.52	1.31 - 1.75	< 0.01
Multivariable 2 ^d^ (Time variant BMI)	0.94	0.78 - 1.13	0.53	1.78	1.55 - 2.05	< 0.01	NA	NA	NA
Stratified ^c^									
*Gender*									
Men	1.03	0.80-1.33	0.82	1.55	1.25-1.92	< 0.01	1.47	1.17-1.85	< 0.01
Women	1.07	0.83-1.37	0.61	1.78	1.47-2.14	< 0.01	1.59	1.32-1.91	< 0.01
*Health insurance*									
Third payer	1.10	0.91-1.32	0.32	1.84	1.59-2.12	< 0.01	1.64	1.41-1.90	< 0.01
State insurance	0.83	0.39-1.77	0.63	0.16	0.1-0.30	< 0.01	0.68	0.34-1.37	0.28
*Early onset DM*									
Diagnosis < 45 y	0.95	0.48-1.84	0.87	2.01	0.93-4.36	0.08	1.72	1.48-1.99	< 0.01
Diagnosis ≥45 y	0.75	0.62-0.90	< 0.01	1.34	1.16-1.54	< 0.01	0.60	0.30-1.20	0.15

*Abbreviations*: *OR* odds ratio, *CI* confidence interval, *BMI* body mass index

^a^ Adjusted for age (continuous), gender (men vs women) and body mass index (continuous and fixed)

^b^ Adjusted for age (continuous), gender (men vs women) and body mass index (continuous and time variant)

^c^ Adjusted for age (continuous), gender (men vs women), body mass index (continuous and fixed), ethnicity (indigenous or afro descendant vs. other), health insurance (state insurance or uninsured vs. third payer) and diabetes duration (continuous)

^d^ Adjusted for age (continuous), gender (men vs women), body mass index (continuous and time variant), ethnicity (indigenous or afro descendant vs. other), health insurance (state insurance or uninsured vs. third payer) and diabetes duration (continuous)

In stratified models we did not observe a significant difference by gender. Regarding health insurance, people cover by the state were more likely to achieve a better glycemic control compared with those insured to the third payer, but this effect was only significantly different in CKD. People with obesity and early onset DM had higher odds of having PGC and this effect was significantly different than observed in those diagnosed ≥45 years (Table 2).

**Fig. 3:**
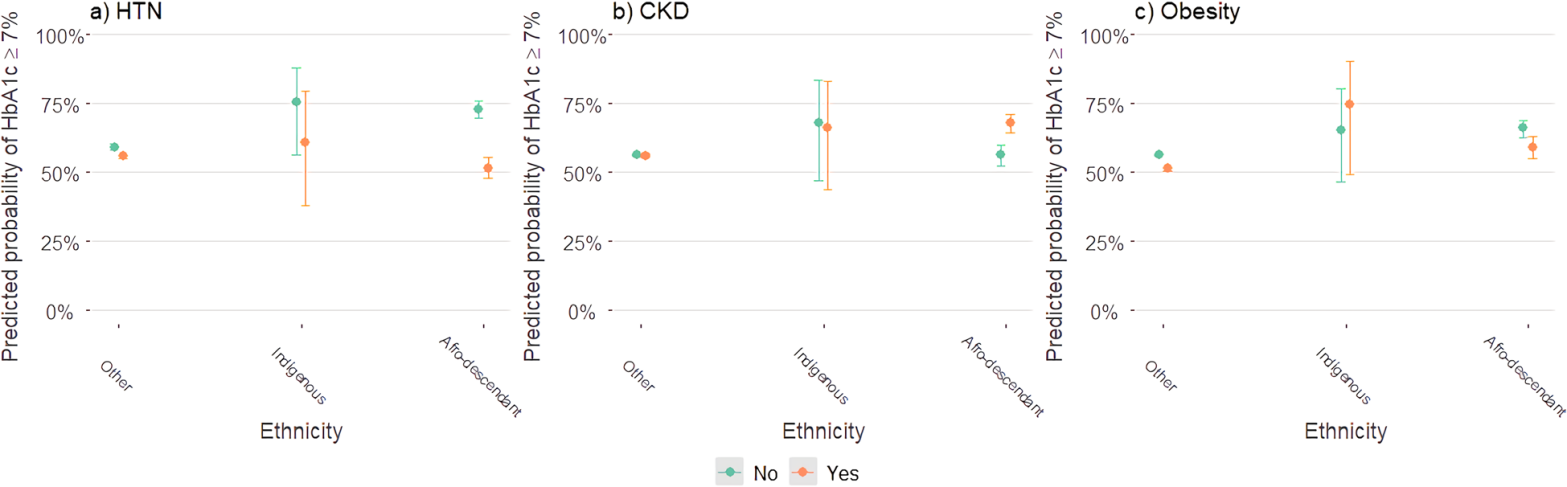
Predicted probabilities of poor glycemic control according to comorbidities and ethnicity, Colombia 2014-2019. Figure 3 shows the predicted probabilities of poor glycemic control according to the presence of comorbidities and ethnicity estimated in a multivariate model adjusted by for age, gender, BMI (only for HTN and CKD), health insurance and diabetes duration. Predicted probabilities of poor glycemic control were significantly lower in afro descendant with HTN (**a**). An opposite association was found in afro descendant with CKD (**b**). In afro descendant with obesity, the association was similar to those with HTN although it was not statistically significant (**c**). Abbreviations: HTN: hypertension, CKD: chronic kidney disease, HbA1c: glycated hemoglobin, BMI: body mass index

When evaluating the interaction between comorbidity and ethnicity, we only identified a statistically significant interaction between each comorbidity and afro-descendants (all *p*-values were < 0.01). Predictive probabilities of having a PGC were significantly lower in afro-descendant with HTN compared with afro descendant without HTN. Otherwise, an inverse effect was found in afro descendant with CKD who had a significantly higher probability of a PGC. Afro descendant with obesity, had a lower probability of a PGC, following the same pattern observed with HTN, however, the effect was not significant (Fig. 3).

### Sensitivity analysis

The associations of interest were consistent with the main models (Additional File 2: Table S2). When analyzing the effect of a BMI ≥ 25 kg/m^2^ on the outcome, we observed the same direction and significance but a lower magnitude in respect of the final model including obesity (OR: 1.26, CI 95%: 1.06-1.49). Similarly, direction and statistical significance remained, although the magnitude decreased when the cut-off point of HbA1c was slightly higher to define the PGC (OR: 1.30, CI 95%: 1.11-1.52). Final models adjusted by early onset DM did not show substantial changes, except for HTN in which direction of the association was inverse although it remained non-significant. Therefore, we hypothesized a potential modifying effect and estimated stratified models as previously described.

## Discussion

This study evaluated the effect that common diabetes associated comorbidities (CKD, HTN and obesity) have on glycemic control among a Colombian adult population living with DM. Longitudinal, non-adjusted trend of HbA1c over time showed a better glycemic control in people with HTN, CKD and non-obese. However, when estimating multivariate models, an inverse effect was found, and people living with diabetes and CKD or obesity had significantly higher odds of having a PGC compared to those without the comorbidities. Whereas a non-significant association was found between HTN and a PGC.

Our study showed that CKD was associated with 78% increased odds of having a PGC. Taking into account that a less strict goal may be recommended in patients living with DM and advanced comorbid diseases [25, 31], we conducted a sensitivity analysis with a less strict threshold (PGC as HbA1c ≥8.0%). Even with this flexible target, the findings were consistent. Patients living with DM and CKD had an increase likelihood of PGC when compared to those without CKD even with a less stringent goal.

Interestingly, we found statistical heterogeneity in the effect of CKD on PGC by health insurance with 84% decreased odds of PGC in people under state insurance whereas third payer affiliates maintained the same trend of the general findings. Previous studies have evaluated the effect of health insurance on glycemic control [32, 33]. A cross-sectional study in Mexico showed that public health affiliates were more likely to have a better glycemic control than uninsured subjects [32]. Similar findings were described by Zhang et al. in the USA where uninsured subjects were more likely to have HbA1c > 9% [33]. In Colombia, both types of insurance have similar access to healthcare services and medications [34], complicating comparisons with other health systems. A possible explanation to our findings could be a higher proportion of stage 5 CKD in the state insurance compared with third payer affiliates (10% versus 1.81%) since the precision of HbA1c reduces with advanced CKD stages and this can lead to underestimation of glycemic levels [31, 35]. Furthermore, when evaluating the interaction between CKD and ethnicity, we found that afro descendants living with DM and CKD also had an increased probability of PGC compared to those without CKD.

Our overall findings are consistent to those found by De Cosmo et al. where CKD (defined as low GFR, high albuminuria or both) was associated with a lower probability of achieving the HbA1c target established in a large Italian population living with DM [36]. However, others have reported that people living with DM and CKD had lower odds of having suboptimal glycemic control [37]. It is important to note that despite the previously described limitations, HbA1c is still considered the standard for glycemic follow-up in patients with CKD [31, 38] thus giving relevance to our results. These findings should increase the concern of clinicians taking care of patients with kidney dysfunction given the consequences of a PGC have on DM patients with CKD. A meta-analysis of patients living with DM treated with hemodialysis described an increased risk of mortality in people with HbA1c ≥8.5% when compared to lower levels (6.5-7.4%) [39]. Also, Kuo et al. observed that HbA1c levels > 9% were associated with an increased risk of end stage renal disease and mortality in patients living with diabetes and CKD stages 3-4 [40].

Similar to the CKD effect on PGC, we observed that obese people were 52% more likely to have a PGC than those without obesity. When a lower threshold was used (≥25 kg/m^2^), the results followed a similar trend, but magnitude decreased to 26%. Together, these results indicate that an elevated BMI increases the risk of PGC in patients living with DM. Similar conclusions have been described in previous studies [37, 41]. A cross sectional study in Iranian population living with diabetes found an association between obesity and poor glycemic control [41]. Furthermore, Bae et al. describe an increased probability of suboptimal glycemic control when overweight or obesity was present in patients living with DM in the USA [37]. Nevertheless, Chetoui et al. did not found an association between overweight/obesity and poor glycemic control in a Moroccan population living with type 2 DM [12]. In a therapeutically point of view, Gummesson et al. stated that weight reduction in overweight and obese patients living with type 2 DM is consistently followed by a reduction on HbA1c levels [42].

Moreover, when evaluating the effect of comorbidities on PGC stratified by age of diagnosis (< 45 years and ≥ 45 years), we found that people with obesity and early onset DM had higher odds of having PGC. These findings are of interest since previous studies have identified a high prevalence of obesity in patients with early onset DM [43, 44]. Additionally, Gopalan et al. described that patients with a diagnosis at 21-44 years had higher initial HbA1c levels and lower odds of achieving glycemic control after a 1 year follow up compared with those diagnosed at 45-64 years [45]. Therefore, these findings support the importance of early-onset DM to further implement specific cost-benefit strategies within this high-risk group to avoid the long-term deleterious complications of PGC.

Some authors have described the presence of HTN as a predictor of poor glycemic control [17, 41]. However, we did not find a significant association between HTN and PGC. Our results are consistent with those of Badedi et al. describing no association between HTN and HbA1c [46]. Though, this was different when evaluating the interaction between ethnicity and HTN. We found that afro descendants living with DM and HTN had a lower predictive probability of PGC than those without HTN; other ethnicities did not have a significant association. Various authors have described an association between ethnicity and glycemic control [10, 11]. A cross sectional study in the USA, found that non-Hispanic blacks and Mexican Americans when compared to non-Hispanic whites had lower odds of adequate glycemic control [11]. Additionally, the same study described that the proportion of patients with three comorbidities (HTN, Hyperlipidemia and obesity) achieving glycemic control (HbA1c < 7.0%) changed according to their ethnicity: 22.8, 11.4 and 0% of non-Hispanic whites, non-Hispanic blacks and Mexican Americans, respectively [11].

High prevalence of PGC could negatively impact health systems. In people living with DM, higher HbA1c levels were associated with an increase in the rate of DM-related hospital admissions [47]. Following a similar trend, Gil et al. evaluated the effect of uncontrolled DM (HbA1c ≥ 7.5%) on health care utilization and found an increase in the number of general practitioner and specialist visits as well as hospital length of stay among male patients living with DM [48]. Furthermore, a cross-sectional study in Spain described that people with PGC had a higher total health-related costs including medication and hospitalization costs when compared to those controlled [49]. The above suggests that a higher proportion of people with PGC may significantly increase the burden on healthcare systems, with a worse impact in low-and-middle-income countries. Thus, our results should encourage public health decision-making for early identification, increased access to health care, and surveillance in subjects living with DM at a higher risk of PGC due to the coexistence of obesity or CKD.

This study has important strengths. First of all, this report reflects a national scope since data come from the largest registry of people with CKD in the country. By including people with different comorbidities, our results are representative of the Colombian population living with DM who receive services within the framework of the health system. Furthermore, a longitudinal approach was used and the effect of repeated measures during the follow up was considered in the final models. This differs from the commonly used cross sectional analysis in studies evaluating factors influencing glycemic control. On the other hand, the use of HbA1c has been well described to evaluate long term glycemic control and because we only studied people with a new DM diagnosis, changes in glycemic control over time are meaningful for decision making process at a clinical and organizational level.

Some limitations should be considered when interpreting our findings. There is a clear effect of medication and non-pharmacological treatment on prospective glycemic control. Nonetheless, we were unable to adjust for treatment because these data were unavailable so residual confusion cannot be ruled out. Following the same line, although some sociodemographic factors such as educational level, income and urban/rural areas have been associated with PGC, we did not evaluate their effects because these variables are not available in the NRCKD. Moreover, the NRCKD is a passive registry and reporting process is performed by health care insurers; this can lead to underreporting and despite it is minimal, it must be declared. Linked to the above, data on variables are likely subjected to measurement error. Nevertheless, its impact was limited through the data monitoring process. In any case this error would be non-differential and effect estimations tend to the null.

## Conclusions

In this adult population living with DM, patients with concomitant CKD or obesity had an increased likelihood of PGC. This supports the importance of weight reduction in diabetes treatment; as well as the increased surveillance and treatment needed in the CKD population. Our findings are based on data from real-world clinical practice thus they should be helpful for public health entities in decision making process. The strategies should be directed in improving access to healthcare, surveillance and treatment in these patients given the detrimental effects of PGC. Whether the presence of HTN modifies the probability of developing a PGC could not be clarified in this study; this effect was only modified in afro descendant ethnicity where HTN decreased the odds of a PGC. Further studies evaluating the effect of pharmacological and lifestyle interventions as well as sociodemographic factors such as educational level, socioeconomic status, or living area (rural versus urban) on the association of interest are needed. The effect of early-onset DM on the increased likelihood of having PGC is noteworthy; hence, further research that evaluates the long-term effect of optimal glycemic levels on the risk of DM-associated complications and mortality is needed.

## Supplementary Information

**Additional file 1. Table S1. **Distribution of Colombian departments by region*. 

**Additional file 2. Table S2. **Multivariate-adjusted odds of poor glycemic control according to sensitivity analysis criteria, Colombia 2014-2019**.**

## Data Availability

The datasets generated and/or analysed during the current study are not publicly available due to that they are owned by the Colombian health system but are available from the corresponding author on reasonable request.

## Abbreviations

ASD: Absolute Standard Differences
BMI: Body Mass Index
CKD: Chronic Kidney Disease
CKD-EPI: Chronic Kidney Disease Epidemiology Collaboration
DM: Diabetes Mellitus
GFR: Glomerular Filtration Rate
HbA1c: Hemoglobin A1c
HTN: Hypertension
IQR: Interquartile range
NRCKD: National Registry of Chronic Kidney Disease
PGC: Poor Glycemic Control SD: Standard deviation
