# Supplementary material for: The effect of comorbidities on glycemic control among Colombian adults with diabetes mellitus: a longitudinal approach with real-world data
Source: BMC Endocr Disord. 2021 Jun 26;21:128. doi: 10.1186/s12902-021-00791-w (PMC8235812; doi:10.1186/s12902-021-00791-w)
Supplement: Supplementary file 1 — Additional file 1. Table S1. Distribution of Colombian departments by region*. [file 12902_2021_791_MOESM1_ESM.docx]

**Additional File 1**

**Table S1. Distribution of Colombian departments by region***

| **Region** | **Colombian departments** |
| --- | --- |
| Bogotá, D.C. | Bogotá, D.C. (Capital city) |
| Central | Antioquia, Caldas, Caquetá, Huila, Quindío, Risaralda and Tolima |
| Eastern | Boyacá, Cundinamarca, Meta, Norte de Santander and Santander |
| Caribbean | Atlántico, Bolívar, Cesar, Córdoba, La Guajira, Magdalena, San Andrés, Providencia y Santa Catalina and Sucre |
| Pacific | Cauca, Chocó, Nariño and Valle del Cauca |
| Amazonian | Amazonas, Arauca, Casanare, Guainía, Guaviare, Putumayo, Vaupés and Vichada |

***Reference:** Departamento Administrativo Nacional de Estadística (DANE). Cuentas departamentales, Producto Interno Bruto por departamento 2019 preliminar. 2020;:7–8. https://www.dane.gov.co/files/investigaciones/pib/departamentales/B_2015/Bol_dptal_2019preliminar.pdf. Accessed 10 Nov 2020
