# Supplementary material for: The effect of comorbidities on glycemic control among Colombian adults with diabetes mellitus: a longitudinal approach with real-world data
Source: BMC Endocr Disord. 2021 Jun 26;21:128. doi: 10.1186/s12902-021-00791-w (PMC8235812; doi:10.1186/s12902-021-00791-w)
Supplement: Supplementary file 2 — Additional file 2. Table S2. Multivariate-adjusted odds of poor glycemic control according to sensitivity analysis criteria, Colombia 2014-2019. [file 12902_2021_791_MOESM2_ESM.docx]

**Additional File 2**

**Table S2. Multivariate-adjusted odds of poor glycemic control according to sensitivity analysis criteria, Colombia 2014-2019**

| **Model** | **Chronic kidney disease^5^** | | | **BMI ≥ 25 kg/m^2^** | | |
| --- | --- | --- | --- | --- | --- | --- |
|  | **OR** | **95% CI** | ***p*-value** | **OR** | **95% CI** | ***p*-value** |
| Age-adjusted | 1.35 | 1.17 - 1.57 | <0.01 | 1.12 | 0.95 - 1.32 | 0.16 |
| Multivariable 1 ^1^ (Fixed BMI) | 1.35 | 1.17 - 1.56 | <0.01 | 1.12 | 0.95 - 1.32 | 0.16 |
| Multivariable 1 ^2^ (Time-variant BMI) | 1.39 | 1.20 - 1.61 | <0.01 | NA | NA | NA |
| Multivariable 2 ^3^ (Fixed BMI) | 1.27 | 1.09 - 1.48 | <0.01 | 1.26 | 1.06 - 1.49 | 0.01 |
| Multivariable 2 ^4^ (Time-variant BMI) | 1.30 | 1.11 - 1.52 | <0.01 | NA | NA | NA |

^1^ Adjusted for age (continuous), gender (men vs women) and body mass index (continuous and fixed).

^2^ Adjusted for age (continuous), gender (men vs women) and body mass index (continuous and time-variant).

^3^ Adjusted for age (continuous), gender (men vs women), body mass index (continuous and fixed), ethnicity (indigenous or afro descendant vs. other), health insurance (state insurance or uninsured vs. third payer) and diabetes duration (continuous).

^4^ Adjusted for age (continuous), gender (men vs women), body mass index (continuous and time-variant), ethnicity (indigenous or afro descendant vs. other), health insurance (state insurance or uninsured vs. third payer) and diabetes duration (continuous).

^5^ Poor glycemic control was defined by a HbA1c ≥ 8.0%.

Abbreviations: OR: odds ratio, CI: confidence interval, BMI: body mass index
